# A Model Iron Gall Ink: An In-Depth Study of Ageing Processes Involving Gallic Acid

**DOI:** 10.3390/molecules27238603

**Published:** 2022-12-06

**Authors:** Adele Ferretti, Francesca Sabatini, Ilaria Degano

**Affiliations:** 1 Department of Chemistry and Industrial Chemistry, University of Pisa, Via G. Moruzzi 13, 56124 Pisa, Italy; 2 Institute of Chemical Science and Technologies “G. Natta” (CNR-SCITEC), Via Elce di Sotto 8, 01628 Perugia, Italy

**Keywords:** iron gall inks, gallic acid, liquid chromatography, tandem mass spectrometry, ageing

## Abstract

Iron gall inks have been among the most used writing materials after carbon black, thus representing an important element of the historical and artistic heritage of our society. Crucially, the preservation of manuscripts and drawings is influenced by the presence of these inks, leading to conservation issues related to paper degradation and text fading. Besides all the advances obtained in paper conservation, the study of iron gall ink’s behaviour and ageing is still an important topic, which requires investigation through an accurate molecular characterisation to produce reliable models. In the present work a micro-destructive method based on liquid chromatography techniques (HPLC-DAD and HPLC-ESI-Q-ToF) has been optimised starting from a model gallic acid-based ink. An in-depth study of the behaviour of the ink in time was performed by natural and artificial ageing tests, monitored by colorimetry, showing the autoxidation of gallic acid to ellagic acid in the prepared mock-ups. The effect of relative humidity on ageing processes was also evaluated, allowing us to determine different intermediates depending on the environmental conditions. Finally, the analytical method developed was then successfully applied for investigating 19th–20th century historical ink samples, where one of the identified ageing markers was detected, besides the expected gallic and ellagic acids.

## 1. Introduction

Inks are liquid, semi liquid or solid materials used for writing, painting or drawing on a support [1]. Chemically, they can be classified as complex mixtures composed of a liquid vehicle, dyes and/or pigments and numerous additives [2,3]. Starting from the Middle Ages and up to the last century (12th–20th century) [4,5,6], *iron gall inks* were elected as the inks *par excellence* given their intense black colour. Recipes for obtaining these inks are widely described in ancient treatises, which mention the use of polyphenols extracted from oak galls, containing hydrolysable gallotannins in combination with green vitriol (FeSO_4_·7H_2_O) and gum arabic, and extensively reported upon in the scientific literature [7,8]. Over the centuries, ink’s recipes have undergone changes, leading to the introduction of new materials and procedures in the late 19th and early 20th century. The chemical knowledge acquired through fundamental and applied research allowed the manufacturers to improve polyphenols-based inks, developing the so-called “unoxidised” and “oxidised” iron gall formulations. Amongst the several recipes published, particular attention was paid to the *gallic acid ink,* introduced by Reid and Dieterich in the 19th century. In this recipe, the ink was prepared from an oxidised gall’s decoction in order to promote the hydrolysis of gallotannins to gallic acid and obtain a molecular profile mainly characterised by gallic acid [9]. Gallic acid (3,4,5-trihydroxy-benzoic acid) is a polyphenol widely present in plants and fruits [10]. Besides its use as a dye, this phenolic acid is extensively applied in the pharmaceutical and food industries for its anti-oxidant, anti-microbial, anti-inflammatory and anti-cancer properties [10,11,12,13,14]. Conversely, many conservation issues arise from the presence of iron gall inks in manuscripts and drawings, such as a conspicuous fading of the text and paper acidification and degradation [7]. The influence of iron gall inks on paper degradation has been widely investigated in the last years, mainly taking into account the role of iron in catalysing Fenton-type reactions [15,16,17,18,19]. X-Ray Fluorescence [20] and Proton Induced X-Ray Emission spectroscopies [19,21] were useful for investigating the spatial distribution of iron compounds in relation to the degradation of ancient manuscripts. Furthermore, Mossbauer [22,23] and X-Ray Absorption Near Edge Structure spectroscopies [24,25,26] enabled the determination of Fe^2+^/Fe^3+^ ratio in iron gall inks, providing a powerful tool for evaluating the damage potential of the ink on manuscripts and drawings. Meanwhile, Fourier-Transform Infrared spectroscopy enabled a foregrounding of the effect of different parameters (e.g., gallic acid concentration, catalytic effect of iron and copper ions) on paper degradation [27,28,29]. The fading issue of iron gall inks has been widely discussed over the years: micro-fading [30] and colour measurements [31] proved the low photosensitivity of these inks. Recent advances have been made by Liu Y. et al. [32], who have demonstrated that the environmental parameters that most influence the discoloration of iron gall inks are the relative humidity and the oxygen concentration.

In spite of wide interest in the topic, to date the effects of ageing and of environmental parameters on iron gall ink’s molecular profile have not yet been fully investigated. There is a general consensus on the main ageing mechanism involving the hydrolysis of gallotannins and ellagitannins [9]. However, the complexity of the whole tannin profile and the many variables related to the use of different raw materials or recipes complicates any in-depth study of ageing processes involving gallic acid, as demonstrated by the recent paper by Teixeira et al. [5]. In the present work an analytical procedure based on High Performance Liquid Chromatography coupled with diode array and tandem mass spectrometric detectors (HPLC-DAD-MS^2^) for analysing iron gall inks has been developed. In order to establish the best experimental conditions, both the sample pre-treatment and the chromatographic method were optimised starting from a simplified iron gall ink’s formulation, prepared using standard gallic acid and iron (II) only and gum arabic as vehicle [33]. The optimised protocol was applied to an in-depth study of the role played by gallic acid in the iron gall ink’s ageing, considering both natural and artificial ageing in different environmental conditions. The analytical method developed was then successfully applied for determining the composition of historical ink formulations, dated to the 19th–20th century, starting from micro-quantities of the sample.

## 2. Results and Discussion

### 2.1. Method Optimisation

#### 2.1.1. Chromatographic Method Optimisation

**Figure 1:**
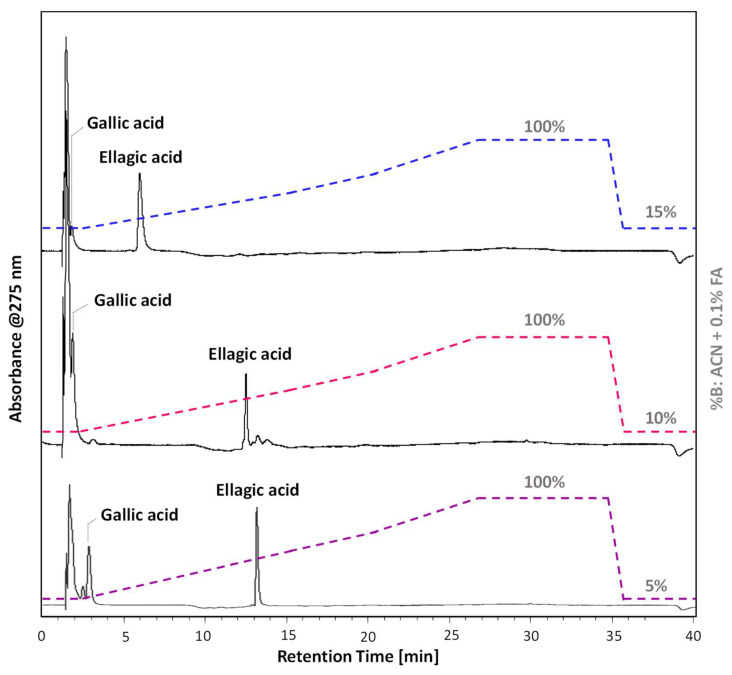
HPLC-DAD chromatograms (extracted at 275 nm) of standard gallic and ellagic acid solution obtained with an initial gradient of: 85% A—15% B (blue gradient), 90% A—10% B (fuchsia gradient), 95% A—5% B (purple profile). A: H_2_O + 0.1% FA, B: ACN + 0.1% FA. The initial gradient is hold for 2.6 min, then to 50% B in 13.0 min, to 70% B in 5.2 min, to 100% B in 6.2 min and then hold for 8.0 min; re-equilibration took 11 min. All chromatograms are presented in the same scale and are stacked for purposes of clarity.

The optimisation of the chromatographic method for HPLC-DAD and HPLC-ESI-Q-ToF analyses was carried out on a standard solution of gallic and ellagic acid. Three different methods were tested, in which the initial percentage of eluents was changed between 85% A and 15% B, 90% A and 10% B, 95% A and 5% B (A: H_2_O + 0.1% FA, B: ACN + 0.1% FA). In all the conditions tested (Figure 1), the peak due to ellagic acid is well resolved, and eluted at times quite distinct from the dead time (5.9 min, 12.6 min and 13.2 min, respectively).

Nevertheless, an efficient chromatographic separation of gallic acid can be achieved by selecting a starting gradient with higher polarity. Therefore, the chromatographic method using an initial gradient of 95% of A and 5% of B was chosen as the optimal chromatographic method for the analysis of iron gall inks.

#### 2.1.2. Extraction Method Optimisation

**Figure 2:**
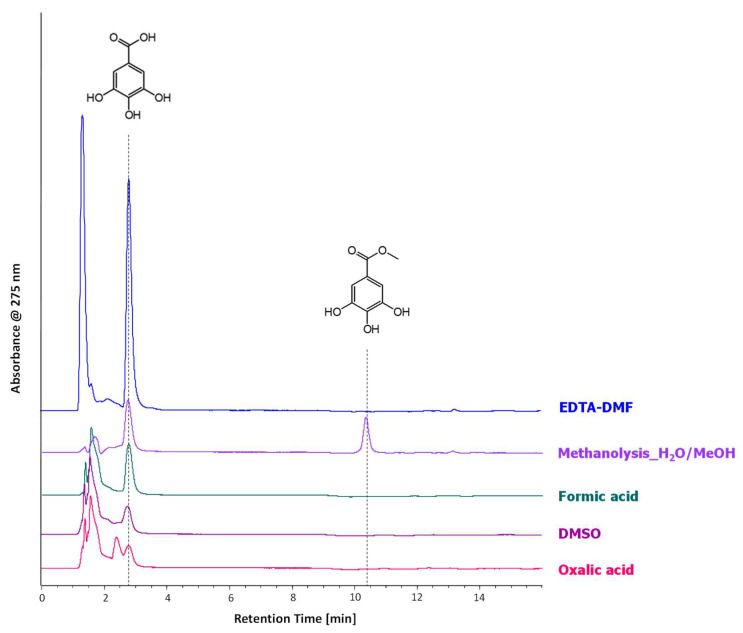
HPLC-DAD chromatograms (275 nm) of the extract obtained from the reference mock-up of model gallic acid ink. EDTA-DMF (blue profile), Methanolysis redissolved in MeOH-H_2_O (lilac profile), Formic acid (green profile), DMSO (purple profile) and Oxalic acid extract (fuchsia profile). All chromatograms are presented in the same scale and are stacked for purposes of clarity.

The optimisation of the extraction method was performed by HPLC-DAD analysis. Samples of the reference mock-ups of model gallic acid ink were collected and subjected to five different extraction methods, as reported in Section 3.5. The chromatograms obtained (Figure 2) indicate that all the treatments tested guarantee the extraction of gallic acid.

Furthermore, methyl-gallate (confirmed by HPLC-ESI-Q-ToF analysis and interpretation of the tandem mass spectrum) was detected in the methanolysis extracts as a by-product due to the hardness of the extraction method used [34,35]. The extraction yield (expressed as mg of dye extracted on g of sample collected) was calculated for each sample pre-treatment (Table S1, Supplementary Materials). The results obtained allow us to identify the EDTA-DMF method and methanolysis with redissolution with MeOH/H_2_O one as the best soft and hard extraction methods, respectively. Nevertheless, the EDTA-DMF treatment shows a higher extraction yield and reproducibility (calculated as CV% on triplicate measurements) than the methanolysis extraction (Table 1). For this reason, the EDTA-DMF treatment was selected as the extraction method for the analysis of iron gall inks on paper support.

### 2.2. Model Gallic Acid Ink

**Figure 3:**
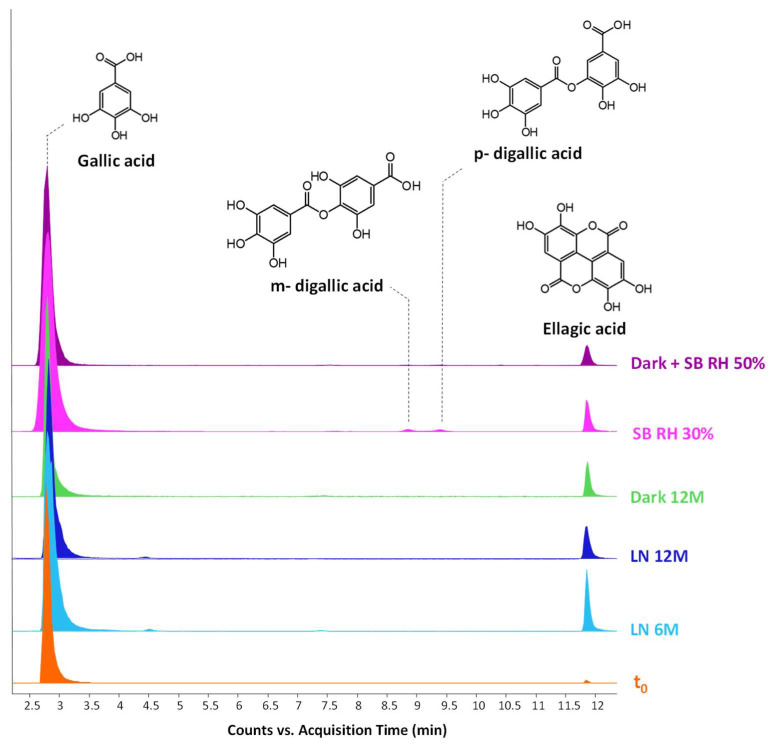
HPLC-ESI-Q-ToF Extract Ion Chromatograms (EIC) of gallic and ellagic acid obtained for the EDTA-DMF extract of the unaged and aged model gallic acid ink. Fresh reference mock-up (orange profile, t_0_), natural light ageing for six months (light blue profile, LN 6M), natural light ageing for twelve months (blue profile, LN 12M), natural ageing in the dark for twelve months (green profile, Dark 12 M), artificial ageing at RH 30% (fuchsia profile, SB RH 30%), artificial ageing at RH 50% (violet profile, Dark+ SB RH 50%). Negative acquisition mode.

The model iron gall ink was prepared on the basis of a simplified recipe [33], inspired by historical recipes and using analytical grade reagents such as standard gallic acid and iron sulphate. The HPLC-DAD (Figure 2) and HPLC-ESI-Q-ToF (Figure 3) analyses performed on the EDTA-DMF extracts show that the main component of the ink is gallic acid.

Additional peaks, absent from the standard solution and corresponding to ellagic acid and m- and p-digallic acids, are also present (in HPLC-DAD ellagic acid is at traces level, visible at 13.1 min in the EDTA-DMF chromatogram in Figure 2). Both isomers of digallic acid have been already determined in iron gall ink formulations [5] or in textiles dyed with oak gall’s polyphenols [9], but never as products of the ageing of standard iron gall ink. Extract Ion Chromatograms (EICs) of gallic and ellagic acid are reported in Figure 3 for the extracts of the ink’s mock-ups unaged and aged in different conditions, normalised towards gallic acid. While gallic acid and ellagic acid were detected in all samples, the isomers of digallic acid were observed only in the mock-up artificially aged at the lowest relative humidity (RH 30%). This is the first instance when ageing products, which were formed starting from gallic acid only, are determined. Ellagic acid may be formed by the auto-oxidation of gallic acid catalysed by iron (III) [36].

**Figure 4:**
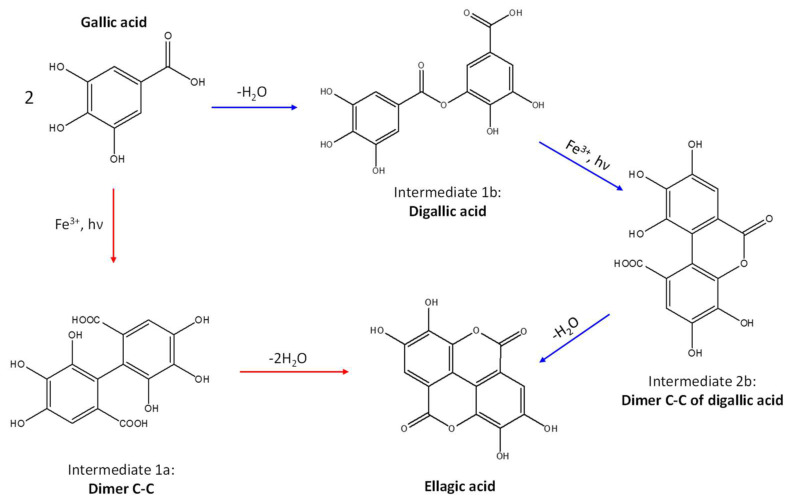
Auto-oxidation of gallic acid to ellagic acid.

Two mechanisms can be hypothesised (Figure 4):

- the dimerisation of gallic acid proceeds through the formation of a C-C bond and subsequent elimination of two water molecules, as already reported in the literature [10,37];
- the dimerisation promotes the formation of digallic acids intermediates, and subsequently the C-C bond is formed and a dehydration reaction takes place.

The experimental evidence suggests two different auto-oxidation mechanisms, whose course is influenced by the relative humidity present in the ageing chamber or in the environment for natural ageing. Indeed, lower humidity promotes the auto-oxidation of gallic acid to digallic acid intermediate, as observed in the chromatogram of artificial ageing at 30% humidity (Figure 3). Instead, higher humidity possibly fosters the auto-oxidation of gallic acid to the C-C dimer intermediate, preventing the formation of digallic acid and its identification in the ageing conditions with RH 50%. Since the presence of iron is clearly a catalyst in the photo-oxidation reaction, the mechanism may be further influenced by other parameters, such as the concentration and oxidation state of iron ions, the presence of other metal ions, and the availability of oxygen and the diffusion of reactive oxygen species (ROS) (i.e., hydroxyl radical (OH^−^), hydrogen peroxide (H_2_O_2_) and superoxide radical (O_2_^−2^)) in the solid sample. Moreover, an increase in the content of ellagic acid was observed with ageing (Figure 3). However, gallic acid does not decrease proportionally to ellagic acid. This may be explained by the parallel formation of different ageing products besides ellagic acid in time, which will be discussed in the next paragraph.

### 2.3. Ageing Degradation Markers

The analytical procedure optimised allowed us to identify numerous ageing and/or degradation products of gallic acid never detected before in the literature for iron-gall inks analysis. Given the ink composition, it is possible to assume that they derive from radical precursors obtained photochemically from gallic acid or ellagic acid or from intermediates of the auto-oxidation mechanism of gallic acid. Specifically, the production of radical precursors could be promoted by sulphate anion [38] (introduced in iron gall inks as *green vitriol*) or by Fenton-type reactions involving iron(II)-iron(III) redox couple. The characterisation of these compounds was performed starting from the exact mass of their molecular ions and from the interpretation of the respective tandem mass spectra obtained with HPLC-ESI-Q-ToF in negative ionisation mode (Figures S3–S15). Table 2 shows the markers identified in the aged reference mock-ups and their hypothesised structures, while the occurrence of each compound in the differently aged ink mock-ups are summarised in Table S2.

**Figure 5:**
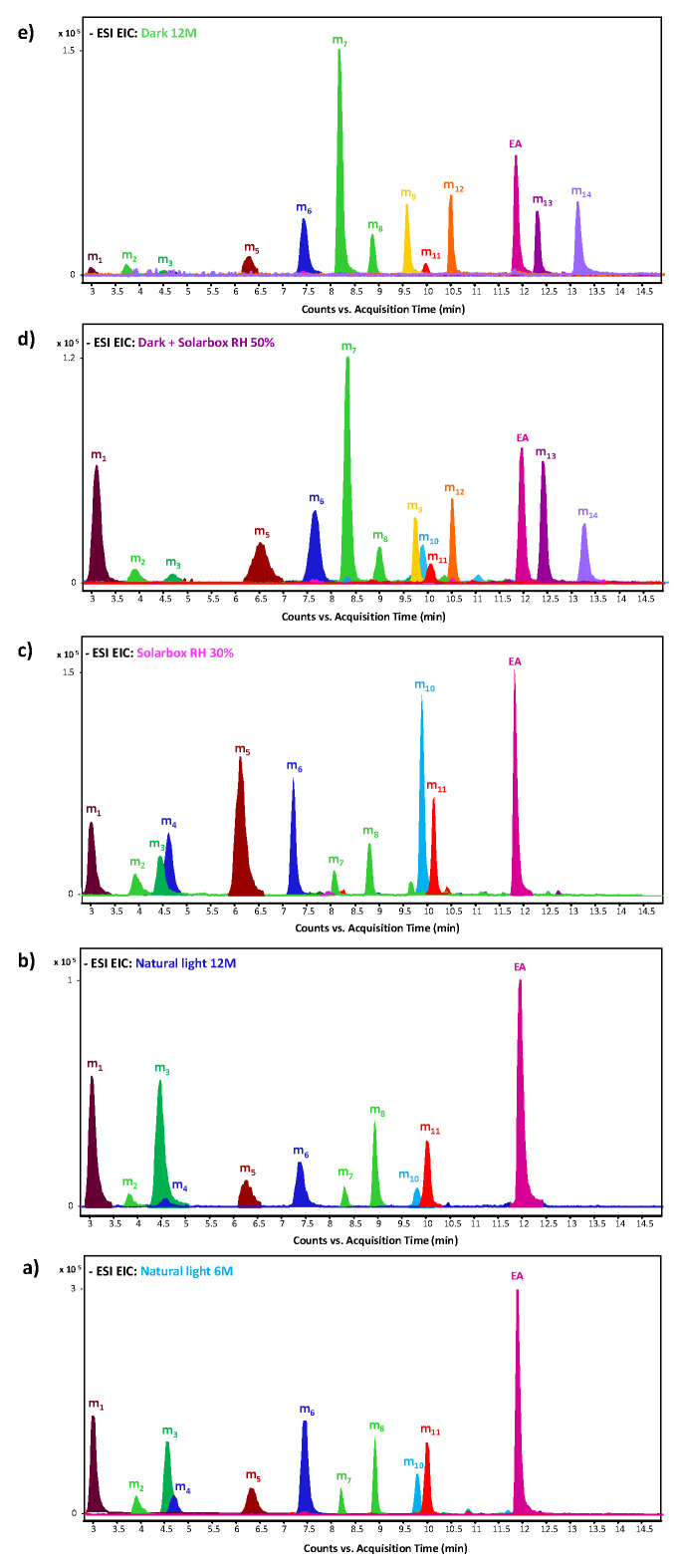
HPLC-ESI-Q-ToF Extract Ion Chromatograms (EIC) of model gallic acid ink degradation products: C_8_H_6_O_7_ (m_1_), C_10_H_6_O_6_ (m_2_,m_7_,m_8_), C_8_H_6_O_6_ (m_3_), C_9_H_8_O_6_ (m_4_,m_6_), C_8_H_6_O_5_ (m_5_), C_15_H_8_O_10_ (m_9_), C_9_H_8_O_5_ (m_10_), C_10_H_8_O_7_ (m_11_), C_17_H_10_O_11_ (m_12_), C_14_H_6_O_8_ (EA, ellagic acid), C_14_H_8_O_7_ (m_13_), C_15_H_8_O_8_ (m_14_) from the extracted aged at: (**a**) Natural light six months; (**b**) Natural light twelve months; (**c**) Solarbox RH 30%; (**d**) Dark + Solarbox RH 50%; and (**e**) Dark twelve months. Negative acquisition mode.

The EIC profiles corresponding to the molecular markers reported in Table 2 and ellagic acid, obtained for the reference ink mock-ups aged under different conditions, are shown in Figure 5.

For all the aged samples, the molecular profile relative to the degradation markers is qualitatively similar but semi-quantitatively different, suggesting that a common degradation mechanism is occurring. Nevertheless, the different ageing condition selected may be responsible for the different relative amounts of the degradation products. Moreover, the ageing conducted in dark conditions allows us to identify four markers not identified in the other mock-ups: C_15_H_8_O_10_, C_17_H_10_O_11_, C_14_H_8_O_7_ and C_15_H_8_O_8_. The hypothesised structures of the degradation markers range from hydroxy and carboxyl substituted gallic acid to two-ring aromatic and heteroaromatic species such as urolithins, which are known metabolic products resulting from the bioconversion of ellagic acid [39,40,41]. Most of these species can be interpreted as intermediate products in the autoxidation of gallic acid to ellagic acid, or as side products of the reactions reported in Figure 4, but they can also be ascribed to the further degradation of ellagic acid under ageing conditions.

### 2.4. Colorimetric Measurements

**Figure 6:**
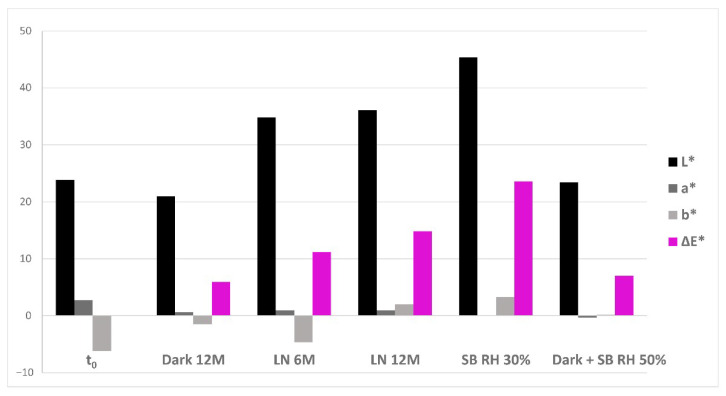
Results of the colorimetric analysis performed on the unaged (t_0_) and aged ink’s reference mock-ups (Dark 12M, LN 6M, LN 12M, SB RH 30%, Dark + SB RH 50%). CIELAB coordinates values are shown in black (L*), grey (a*), dark grey (b*) and fuchsia (ΔE*, calculated respect to t_0_).

Colorimetric measurements enabled us to evaluate the impact of fading mechanism on the aged ink’s mock-ups. The results obtained (Figure 6) show a significant increase in b* in all the tested ageing conditions with respect to t_0_, proving that yellowing occurs in relation to iron gall inks, as already reported in the literature [42,43].

Moreover, a decrease in a* was also determined; however, it does not change significantly if compared to the trends exhibited by the other chromatic coordinates. Concerning the L* parameter, two different trends were observed depending on the ageing conditions. On one side, natural light and solarbox RH 30% ageing caused an increase in brightness, consistent with the light-fading visible to the naked eye. On the other side, natural dark and solarbox RH 50% ageing did not impart significative variations in brightness. The two sets of mock-ups displaying different behaviours in terms of L* changes correspond indeed to references characterised by qualitatively similar molecular profiles of degradation markers, as shown in Section 2.3 and Figure 5. The same trend is observed for ΔE*, calculated with respect to t_0_. In detail, the extracts of the mock-ups aged in solarbox RH 50% and in the dark show ageing markers (m_9_, m_12_, m_13_ and m_14_) that were not identified in the other ageing conditions. Since these degradation products have been characterised as compounds featuring three conjugated cycles, it is possible to hypothesise that they contribute to providing a darker colour to the aged ink’s mock-ups. This could counteract the light-fading mechanism observed under the other ageing conditions. Indeed, the observed ΔE* are all perceptible to the human eye (threshold c.a. 0.8). However, further studies are needed in order to confirm this hypothesis, and to verify whether it applies to traditional iron gall ink’s formulations.

### 2.5. Cases Studies

**Figure 7:**
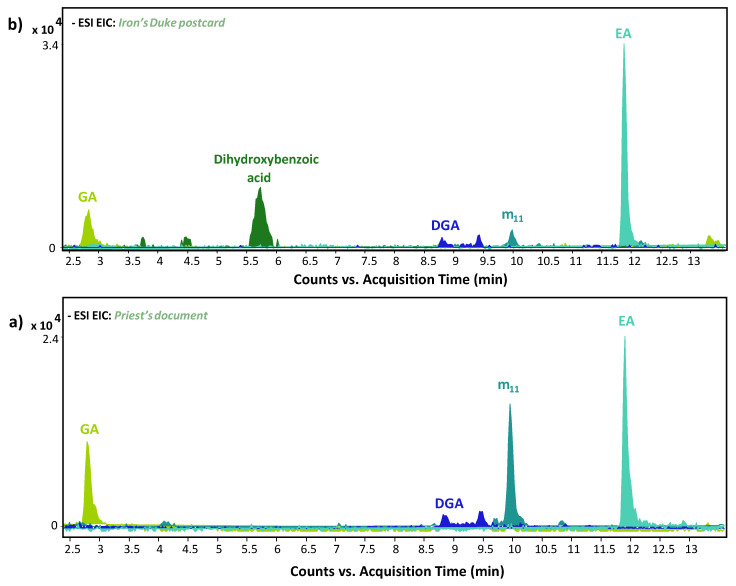
HPLC-ESI-Q-ToF Extracted Ion Chromatogram (EICs) of gallic acid (GA, C_7_H_6_O_5_), digallic acid (DGA, C_14_H_10_O_9_), ellagic acid (EA, C_14_H_6_O_8_), m_11_ (C_10_H_8_O_7_) and dihydroxybenzoic acid (C_7_H_6_O_4_) for Priest’s document (Figure (**a**)) and Iron’s Duke postcard (Figure (**b**)).

The HPLC-ESI-Q-ToF analysis performed on Priest’s document and on Iron’s Duke postcard (Figure 7) highlighted the presence of gallic, ellagic and digallic acids, along with dihydroxybenzoic acid ([M-H]^−^ = 153.019), suggesting the use of an iron gall-based ink. The latter is a known component of tannin materials [9].

Furthermore, the ageing marker of iron gall ink m_11_ ([M-H]^−^ = 239.020) was determined in both samples.

## 3. Materials and Methods

### 3.1. Reagents and Solvents

Gallic acid and ellagic acid were purchased respectively from LabService Analytica (Italy) and Lancaster (UK). The solvents and reagents used for the extraction methods were: ethylenediaminetetraacetic acid disodium salt (EDTA, Fluka, USA), dimethylformamide (DMF; 99.8% purity, J.T. Baker, USA), dimethyl sulfoxide (DMSO; 99.8% purity, J.T. Baker, USA), acetonitrile (ACN; HPLC grade, Sigma Aldrich, St. Louis, MO, USA), oxalic acid dehydrate (99.8% purity, Carlo Erba, Milan, Italy), methanol (MeOH; 99.9% purity, Sigma Aldrich, St. Louis, MO, USA), acetone (Sigma Aldrich, St. Louis, MO, USA), hydrochloric acid (HCl; 37%, Merck, Darmstated, Germany) and formic acid (FA; J.T. Baker, USA). The eluents used for HPLC-DAD and HPLC-ESI-Q-ToF were: water and acetonitrile, both HPLC grade (Sigma Aldrich, St. Louis, MO, USA) for HPLC-DAD analysis and both LC-MS grade (Sigma Aldrich, St. Louis, MO, USA) for HPLC-ESI-Q-ToF analysis. All eluents were added with 0.1% *v/v* FA.

### 3.2. Reference Model Ink

In the present work a model iron gall ink was studied, prepared following the recipe used for the model gallic acid ink in [33]. The solvents and materials used for the model ink’s production were: ferrous sulphate heptahydrate (FeSO_4_·7H_2_O; Analyticals, Carlo Erba, Italy), gum arabic (Kremer-pigmente, Germany) and deionised water. The reference model ink was prepared as follows [33]: 1.2 g of gallic acid and 2.0 g of ferrous sulphate heptahydrate were solubilised in 150 mL and in 5 mL of deionised water, respectively. The two solutions were mixed, and the resulting solution was kept in the dark at room temperature for one week. Afterwards, any solid was filtered from the solution, and 3.0 g of milled gum arabic were added to obtain the final ink.

### 3.3. Reference Mock-Ups and Ageing Tests

Reference mock-ups were prepared casting the model iron gall ink on Whatman filter paper (USA, grade 42, diameter 110 mm, pure cellulose), selected in accordance with the literature as pure cellulose support without any additives [6,17,43]. Each reference mock-up was a semicircle with 4.5 cm radius (approx.). The ink was applied on filter paper with a Pasteur pipette and spread with a spatula, twice (corresponding to c.a. 30 μL/cm^2^). Five mock-ups were prepared: one was used as a starting (t_0_) reference, one was subjected to natural ageing indoor and in the dark, and two were artificially aged (Test 1 and Test 2). Natural light ageing was performed exposing mock-ups to natural indoor light in laboratory conditions (T = 25 ± 1 °C, RH 50%). In order to monitor the ageing steps, samples were collected after six and twelve months from the beginning of the experiment. Instead, natural ageing in the dark was carried out storing the reference mock-up in the dark in laboratory conditions. Artificial ageing was performed using a SolarBox (CO.FO.ME.GRA. Srl, Milan, Italy) equipped with a Xe lamp and an indoor filter. To evaluate the impact of humidity on artificial ageing, two tests were carried out: one with the relative humidity (RH%) at 30% and the other at 50% RH. The values of the selected parameters for artificial ageing are listed in Table S3 (Supplementary Materials).

### 3.4. Historical Samples of Ink Handwriting

**Figure 8:**
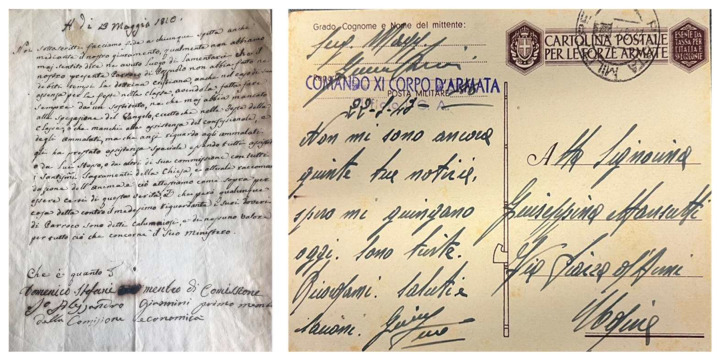
Photo of Iron’s Duke postcard (**right**) and Priest’s document (**left**).

The optimised method was used to analyse two historical ink samples. The Iron’s Duke postcard is a postcard written by an Italian soldier to his family with a black ink and dated 1943; for the analysis, 30 μg of sample were collected. The Priest’s document is a document from 1820 (kindly provided by Dr. Giovanni Casini) written with a black-brown ink. The entire document is characterised by numerous faded spots due to humidity; the non-pristine state of the document allowed us to collect a higher quantity of sample (300 μg) without impairing the readability or the overall appearance of the text. Photos of Iron’s Duke and Priest’s document are shown in Figure 8.

### 3.5. Sample Treatments

Five sample pre-treatments, already reported in the literature mainly for the analysis of dyes in historical textiles, were tested on a few mg of reference samples: (i) addition of 500 μL of DMSO, sonication (ultrasonic bath Sonorex Supra 10P, Bandelin Electronics, Germany) for 30 min at 60 °C; (ii) addition of 500 μL of 0.1% EDTA aqueous solution-DMF (EDTA-DMF, 1:1, *v/v*), sonication for 1 h at 60 °C; (iii) addition of 500 μL of 0.5 M oxalic acid aqueous solution/MeOH/acetone/H_2_O solution (oxalic acid solution, 1:30:40:40, *v/v/v/v*), sonication for 30 min at 60 °C, drying and re-dissolution in 500 μL of DMSO; (iv) addition of 500 μL of formic acid/MeOH solution (1:19, *v/v*), sonication for 30 min at 60 °C, drying and re-dissolution in 500 μL of DMSO; (v) addition of 500 μL of HCl 37%/MeOH solution (1:30, *v/v*), sonication for 60 min at 60 °C, drying and re-dissolution in 500 μL of solvents. For the re-dissolution step of the acidic methanolic treatment, five solvents were tested: DMF, DMSO, MeOH, MeOH/H_2_O (1:1, *v/v*) and ACN/H_2_O (1:1, *v/v*). Prior to HPLC analysis, the supernatant was purified with PTFE syringe filters (4 mm thickness and 0.45 μm pore diameter, Agilent), and then injected in the chromatographic system. For the analysis of the reference inks, 3 mg of reference mock-ups were collected using a micro-hole hand-held paper puncher. The collection step was performed in different points of the surface, to minimise variability due to the inhomogeneity of casting. To evaluate the reproducibility of the detected molecular profiles, some selected reference samples were analysed in duplicates. For the analysis of historical ink samples, 30–300 μg of sample were collected by scratching the surface with a scalpel, where the manuscript showed defects (e.g., ink drops, lacunae in the manuscript, etc.), and treated with 50 μL of 0.1% EDTA aqueous solution-DMF in ultrasonic bath for 1 h at 60 °C. The extracts were filtered and injected in the chromatographic system.

### 3.6. Colorimetric Measurements

A Konica-Minolta Mod. CM-700d with 3 mm spot (diameter), wavelength range 400–700 nm and d:8° geometry for the illuminant/observer was employed. Specular component excluded (SCE) readings were recorded. Three measurements on three different spots were averaged for each unaged and aged ink’s mock-up, converted to CIE 1976 L*a*b* space and processed with Microsoft Excel. The CV% of the three replicates in terms of ΔE was below 5%. The colour difference (ΔE) was calculated by comparing the values obtained for unaged and aged ink’s mock-ups and using the colour difference equation developed by the Society of Dyers and Colourists, Color Measurement Committee (CMC). Colorimetric measurements were also performed on the unaged and aged paper supports; however, no significant variation in terms of ΔE was observed.

### 3.7. HPLC-DAD-MS^2^ Analysis

The HPLC system consists of a PU-2089 quaternary pump (Jasco International Co., Tokyo, Japan) equipped with a degasser, an AS-950 autosampler (Jasco International Co., Tokyo, Japan), an MD-2010 spectrophotometric diode array detector (DAD) (Jasco International Co., Tokyo, Japan). ChromNav software was used to carry out data acquisition and data analysis. The DAD detector operated with spectra acquisition in the range of 200–650 nm every 0.2 s with 4 nm resolution.

For HPLC-ESI-Q-ToF analysis, an HPLC 1200 Infinity, coupled to a Jet Stream ESI-Q-ToF 6530 Infinity detector and equipped with an Agilent Infinity autosampler (Agilent Technologies, Palo Alto, CA, USA), was used. MassHunter^®^ Workstation Software (B.04.00) was used to carry out mass spectrometer control, data acquisition and data analysis. The mass spectrometer operated in ESI ionisation in negative mode and the working conditions were: drying gas N_2_ (purity>98%) temperature 350 °C and 10 L/min flow; capillary voltage 4.5 KV; nebuliser gas pressure 35 psig; sheath gas temperature 375 °C and 11 L/min flow; fragmentor voltage 175 V. High resolution MS and MS/MS spectra were acquired in negative mode in the range 100–1700 m/z at a scan rate of 1.04 spectra/sec (CID voltage 30 V, collision gas N_2_, purity 99.999%). Auto-calibration was performed daily using Agilent tuning mix HP0321 (Agilent Technologies) prepared in acetonitrile.

The chromatographic separation was performed in both systems on an analytical reversed-phase column Poroshell 120 EC-C18 (3.0 × 75 mm, particle size 2.7 μm,) with a pre-column Zorbax (4.6 × 12.5 mm, particle size 5 μm) both Agilent Technologies (Palo Alto, CA, USA). The eluents were A: formic acid (FA 0.1% *v/v*) in LC-MS grade water and B: formic acid (FA 0.1% *v/v*) in LC-MS grade acetonitrile. The flow rate was 0.4 mL/min and the program was 5% B for 2.6 min, then to 50% B in 13.0 min, to 70% B in 5.2 min, to 100% B in 6.2 min and then hold for 8.0 min; re-equilibration took 11 min. During the separation, in both systems, the column was thermostated at 30 °C. The injection volume was 10 μL and 4 μL for the HPLC-DAD and HPLC-MS/MS analysis, respectively.

### 3.8. Quantitative Analysis

Quantitative analysis was performed by HPLC-DAD. Stock and working solutions of gallic and ellagic acid were prepared in DMSO. The concentrations used were 2.5, 5, 10, 20 and 25 ppm. The calibration curves were obtained by integrating the gallic and ellagic acid peak in the HPLC-DAD chromatogram acquired at 275 nm and 365 nm respectively. The calibration curves coefficients obtained by linear regression fitting (y = mx + q) are reported in Table S4 (Supplementary Materials). The standard solutions were analysed by both HPLC instrumentations. Table 3 shows all the parameters useful for the identification of gallic and ellagic acid by HPLC-DAD and HPLC-ESI-Q-ToF analysis.

## 4. Conclusions

In the present work both the chromatographic method and the sample treatment protocol for the analysis of iron gall inks on paper support have been optimised. The devised methodology, based on ultra-sensitive chromatographic and mass spectrometric methods, guarantees the identification of the molecular marker of the dye and its ageing products in ink mock-ups. To the best of our knowledge this is the first time that a model iron gall ink has been used to perform an in-depth study of the ageing mechanism of gallic acid. Regarding our studies, there are two remarkable results that could bring new information to the literature. First, this research enabled us to demonstrate that the auto-oxidation of gallic acid to ellagic acid occurs with the ageing of the model iron gall ink. Furthermore, we verified that the entire mechanism is influenced by environmental parameters during ageing. The impact of relative humidity was evaluated, showing that lower RH% fosters the auto-oxidation mechanism through digallic acid intermediates, while higher RH% favours the mechanism through dimer C-C intermediate. To date, only the hydrolysis of gallotannins and ellagitannins has been taken into account to model the ageing mechanism of iron gall inks. Therefore, our studies could provide the springboard for a new way to investigate ageing phenomena in these inks’ formulations. Second, high resolution tandem mass spectrometry enabled us to identify numerous ageing and/or degradation products of gallic acid never detected before for iron gall inks. Most of these markers can be interpreted as intermediate or side products in the autoxidation of gallic acid to ellagic acid, but they can also be ascribed to the further degradation of ellagic acid. However, these results allow us to widen the database of molecular markers available for the identification of iron gall inks in manuscripts and drawings. Furthermore, colorimetric measurements enabled us to show that four degradation markers (m_9_, m_12_, m_13_ and m_14_) could cause a darkening, which in turn reduces the visible fading of iron gall inks.

The developed protocol was applied to two case studies and, indeed, the marker m_11_ was detected in both, confirming the validity of this compound as an ageing marker of iron gall inks. None of the other markers described were detected in the historical samples, suggesting that their presence was below the detection limit or that they are intermediate ageing products whose fate may be further investigated by prolonging the ageing time of the model ink.

## Figures and Tables

**Table 1:** Extraction yields (mg/g), standard deviation (S) and coefficient of variability (CV%) obtained for the EDTA-DMF and the Methanolysis (H_2_O/MeOH) extract of model gallic acid ink.

Extraction Method	mg/g (Average) of Gallic Acid	S (mg/g)	CV %
EDTA-DMF (0.1% of EDTA in H_2_O/DMF 1:1)	10.4	0.4	4
Methanolysis (HCl/MeOH 1:30) redissolved in H_2_O/MeOH	2.7	0.6	23

**Table 2:** List of compounds identified in the aged reference mock-ups of model gallic acid ink (n.a. = not assigned). The most intense product ions are highlighted in bold.

Ageing Marker	t_R_ (min)	[M-H]^−^	MS^2^	Raw Formula (ppm)	Hypothesised Structure
m_1_	2.9	213.0041	151.002, 123.007, **107.014**	C_8_H_6_O_7_ −0.3	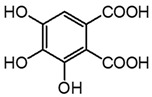
m_2_	3.9	221.0094	177.016, 149.023, 132.018, **121.026**, 109.029, 105.031	C_10_H_6_O_6_ 1.2	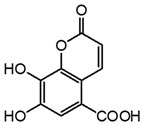
m_3_	4.4	197.0097	152.010, **125.024**, 107.013	C_8_H_6_O_6_ 3.1	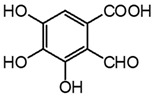
m_4_	4.6	211.0254	167.032, 150.915, 139.040, **124.016**, 109.028	C_9_H_8_O_6_ 3.2	n.a.
m_5_	6.2	181.0139	137.022, **109.029**	C_8_H_6_O_5_ −1.4	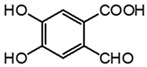
m_6_	7.3	211.0245	167.032, 150.920, 132.904, **124.015**, 107.013	C_9_H_8_O_6_ −1.7	n.a.
m_7_	8.2	221.0093	177.015, 166.859, 148.012, **133.025**, 123.008, 115.016, 103.016	C_10_H_6_O_6_ 0.1	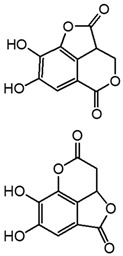
m_8_	8.9	221.0088	**177.015**, 160.014, 149.021, 133.025, 121.029, 105.031	C_10_H_6_O_6_ −1.8	
m_9_	9.5	347.0040	**259.026**, 213.021, 187.041, 109.030	C_15_H_8_O_10_ −1.8	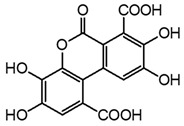
m_10_	9.7	195.0303	150.031, 136.017, **123.044**, 108.021	C_9_H_8_O_5_ 1.9	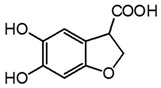
m_11_	9.9	239.0169	193.009, 167.035, 152.011, **125.024**, 107.013	C_10_H_8_O_7_ −3.5	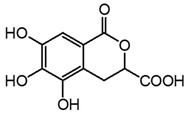
m_12_	10.5	389.0145	**301.037**, 258.018, 165.020, 123.010	C_17_H_10_O_11_ −1.2	n.a.
m_13_	12.3	287.0198	243.025, 225.015, **197.020**, 171.042, 159.041, 143.047	C_14_H_8_O_7_ 0.3	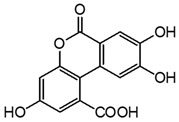
m_14_	13.2	315.0145	287.021, 271.024, **243.031**, 215.036, **197.025**, 187.040, 171.046, 159.047, 143.050	C_15_H_8_O_8_ 0.5	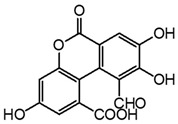

**Table 3:** Parameter for HPLC-DAD and HPLC-ESI-Q-ToF identification of gallic and ellagic acid. The most intense product ions are highlighted in bold.

Analyte	Chemical Structure	t_R_ (min) HPLC-DAD	λ_max_ (nm)	t_R_ (Min) HPLC-MS	MS ([M-H]^−^)	MS^2^
Gallic acid	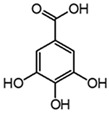	2.8	271	2.8	169.014	**125.024**, 107.014
Ellagic acid	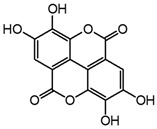	13.1	255, 367	11.8	300.997	**300.994**, 283.993, 257.006, 229.014, 185.026, 145.029

## Data Availability

The data that support the findings of this study are available from the corresponding author, I.D., upon reasonable request.

## Supplementary Materials

The following supporting information can be downloaded at: https://www.mdpi.com/article/10.3390/molecules27238603/s1, Table S1: Extraction yields obtained for gallic acid extract from model gallic acid inks mock-ups; Table S2: Molecular markers identified in the aged and unaged model gallic acid ink mock-ups.; Table S3: Parameters used for artificial ageing tests; Table S4: Calibration curves parameters for gallic and ellagic acid in the HPLC-DAD system; Figure S1: Tandem mass spectrum of gallic acid; Figure S2: Tandem mass spectrum of ellagic acid; Figure S3–S15: Tandem mass spectra of m_1_–m_14_ with hypothesised structures.

## Sample Availability

Samples of all reference materials are available from the authors upon motivated request.
