# Supplementary material for: A Model Iron Gall Ink: An In-Depth Study of Ageing Processes Involving Gallic Acid
Source: Molecules. 2022 Dec 6;27(23):8603. doi: 10.3390/molecules27238603 (PMC9735674; doi:10.3390/molecules27238603)
Supplement: Supplementary file 1 [file molecules-27-08603-s001.zip › molecules-2030471-supplementary.pdf]

SUPPLEMENTARY MATERIALS for

A model iron gall ink: an in-depth study of ageing processes involving gallic acid

Adele Ferretti[1], Francesca Sabatini[2] and Ilaria Degano*[1]

[1] Department of Chemistry and Industrial Chemistry, University of Pisa, Via G. Moruzzi 13, 56124, Pisa, (Italy);
[2] Institute of Chemical Science and Technologies "G. Natta" (CNR-SCITEC), Via Elce di Sotto 8, 01628, Perugia, (Italy);
* Correspondence: ilaria.degano@unipi.it

- **Table S1**: Extraction yields obtained for gallic acid extract from model gallic acid inks mock-ups;
- **Table S2**: Molecular markers identified in the aged and unaged model gallic acid ink mock-ups;
- **Table S3**: Parameters used for artificial ageing tests;
- **Table S4**: Calibration curves parameters for gallic and ellagic acid in the HPLC-DAD system;
- **Figure S1**: Tandem mass spectrum of gallic acid;
- **Figure S2**: Tandem mass spectrum of ellagic acid;
- **Figure S3-S15**: Tandem mass spectra of m_1-m_{14} with hypothesised structures.

Table S.1: Extraction yields obtained for gallic acid extract from model gallic acid inks mock-ups.

Extraction methods	Extraction yields (mg of dye/g of sample)
**EDTA-DMF** (0.1% of EDTA in H_2O/DMF 1:1)	12.9
**DMSO**	0.9
**Oxalic acid solution** ($C_2H_2O_4$/MeOH/acetone/H_2O, 1:30:30:40)	0.5
**Formic acid solution** (HCOOH/MeOH, 1:19)	0.4
**Methanolysis** (HCl/MeOH 1:30) redissolved in **DMSO**	0.4
**Methanolysis** (HCl/MeOH 1:30) redissolved in **MeOH**	1.0
**Methanolysis** (HCl/MeOH 1:30) redissolved in **DMF**	0.1
**Methanolysis** (HCl/MeOH 1:30) redissolved in **H_2O/ACN** (1:1)	0.7
**Methanolysis** (HCl/MeOH 1:30) redissolved in **H_2O/MeOH** (1:1)	1.8

Table S.2: Molecular markers identified in the aged and unaged model gallic acid ink mock-ups. The markers detected at trace levels are indicated as (tr).

Compound	t_0	LN 6M	LN 12M	Dark 12M	SB RH 30%	Dark + SB RH 50%
**Gallic acid**	x	x	x	x	x	x
Marker 1		x	x	tr	x	x
Marker 2		x	x	tr	x	x
Marker 3		x	x	tr	x	x
Marker 4		x	x		x	
Marker 5		x	x	x	x	x
Marker 6		x	x	x	x	x
Marker 7		x	x	x	x	x
Marker 8		x	x	x	x	x
**m-digallic acid**					x	
**p-digallic acid**					x	
Marker 9				x		x
Marker 10		x	x		x	x
Marker 11		x	x	x	x	x
Marker 12				x		x
**Ellagic acid**	x	x	x	x	x	x
Marker 13				x		x
Marker 14				x		x

Table S.3: Parameters used for artificial ageing tests.

	**Irradiance** (W/m^2)	**Temperature** (°C)	**Relative humidity** (%)	**Time** (weeks)
Test 1	1000	40	30	4
Test 2	550	40	50	8

Table S.4: Calibration curves coefficients for gallic and ellagic acid in the HPLC-DAD system.

**Analyte**	**m (Area)**	**q (Area/mg)**	**R^2**
Gallic acid	7.6×10^4	-3.1×10^4	0.999
Ellagic acid	5.5×10^4	-1.0×10^5	0.993

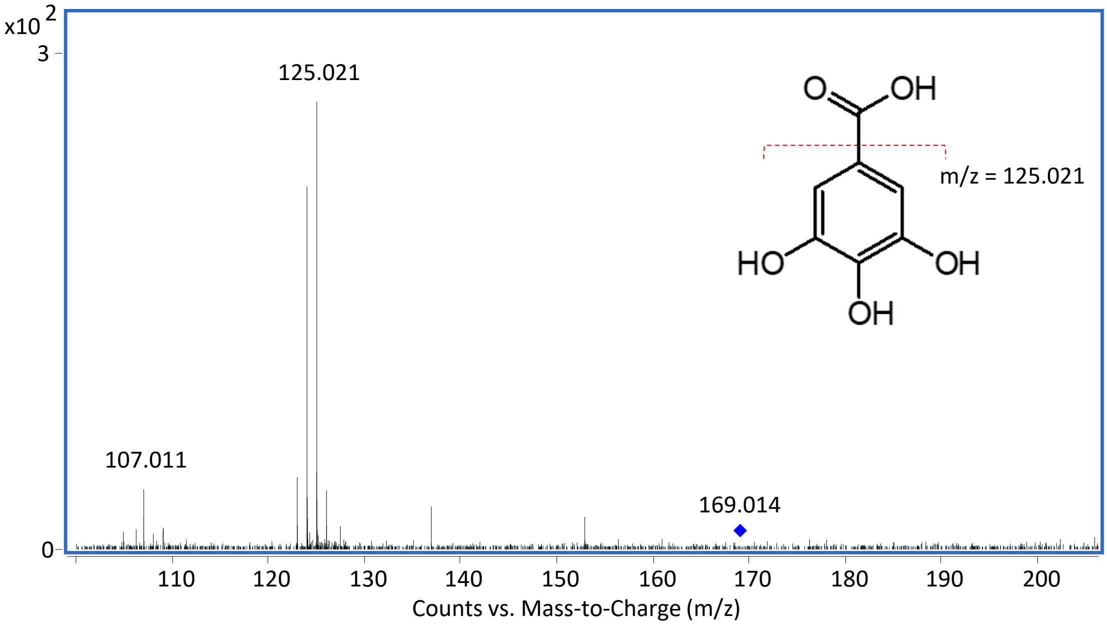

Figure S.1: Tandem mass spectrum of gallic acid (t_R = 2.8 min, $C_7H_6O_5$)

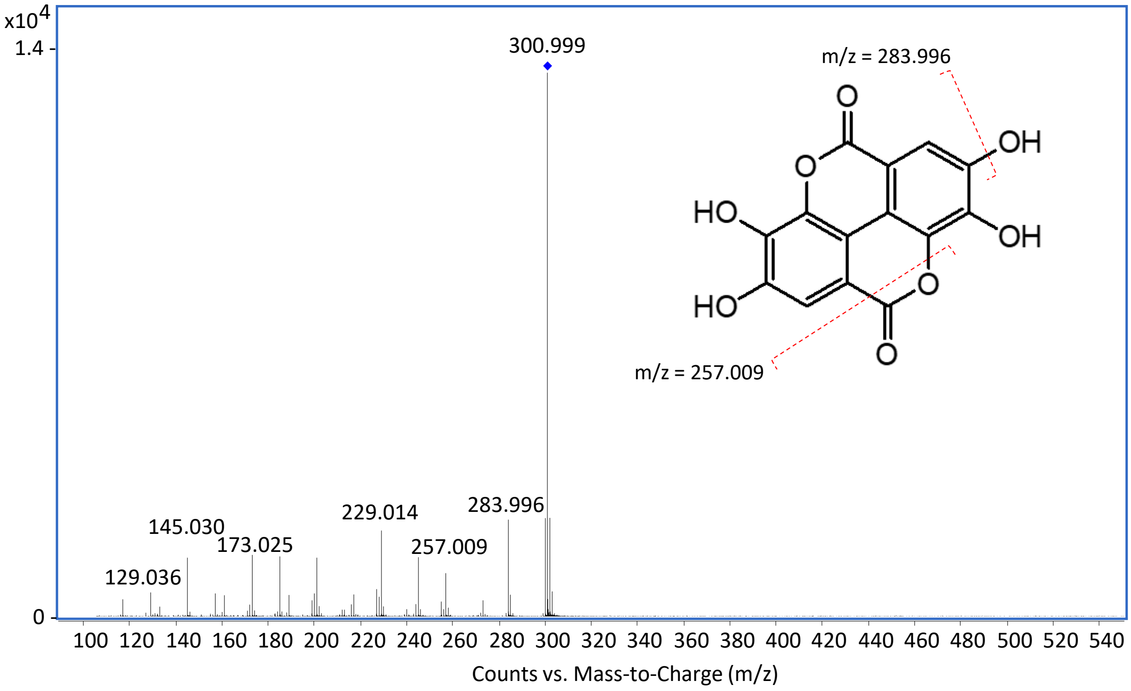

Figure S.2: Tandem mass spectrum of ellagic acid (t_R = 11.8 min, $C_{14}H_8O_6$)

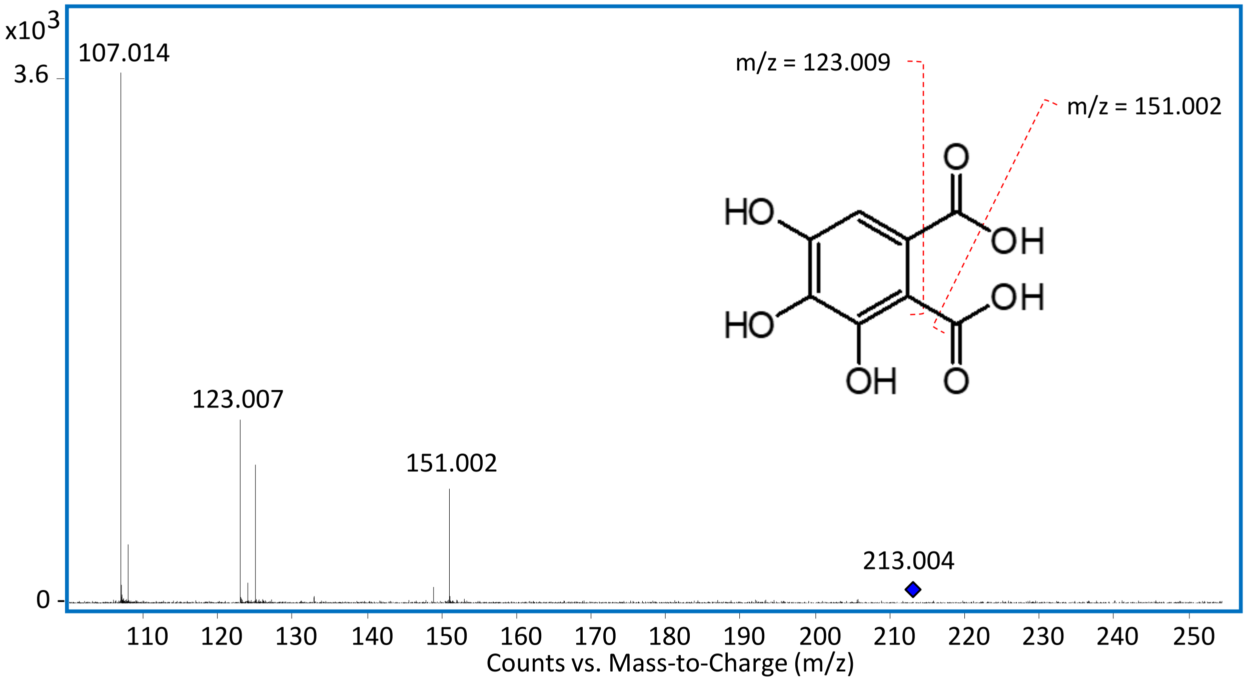

Figure S.3: Tandem mass spectrum of m_1 (t_R = 2.9 min, $C_8H_6O_7$) with hypothesised structure

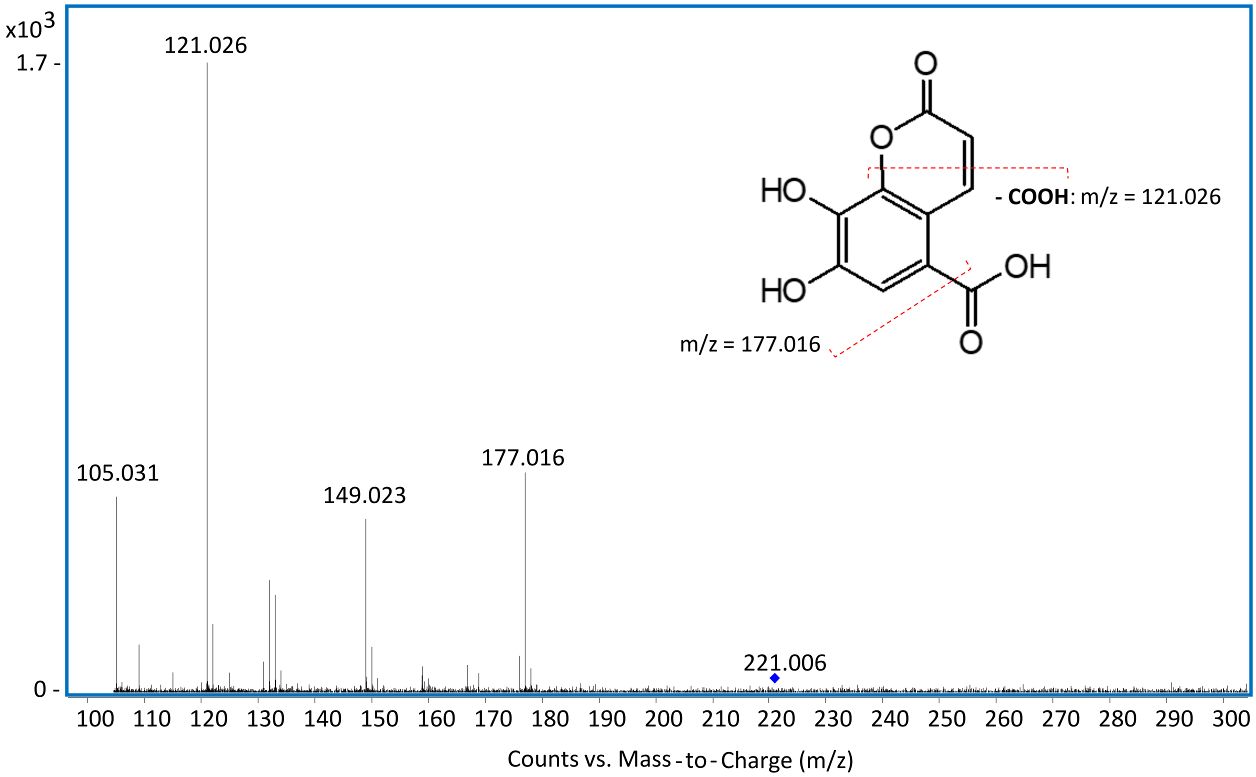

Figure S.4: Tandem mass spectrum of m_2 (t_R = 3.9 min, $C_{10}H_6O_6$) with hypothesised structure

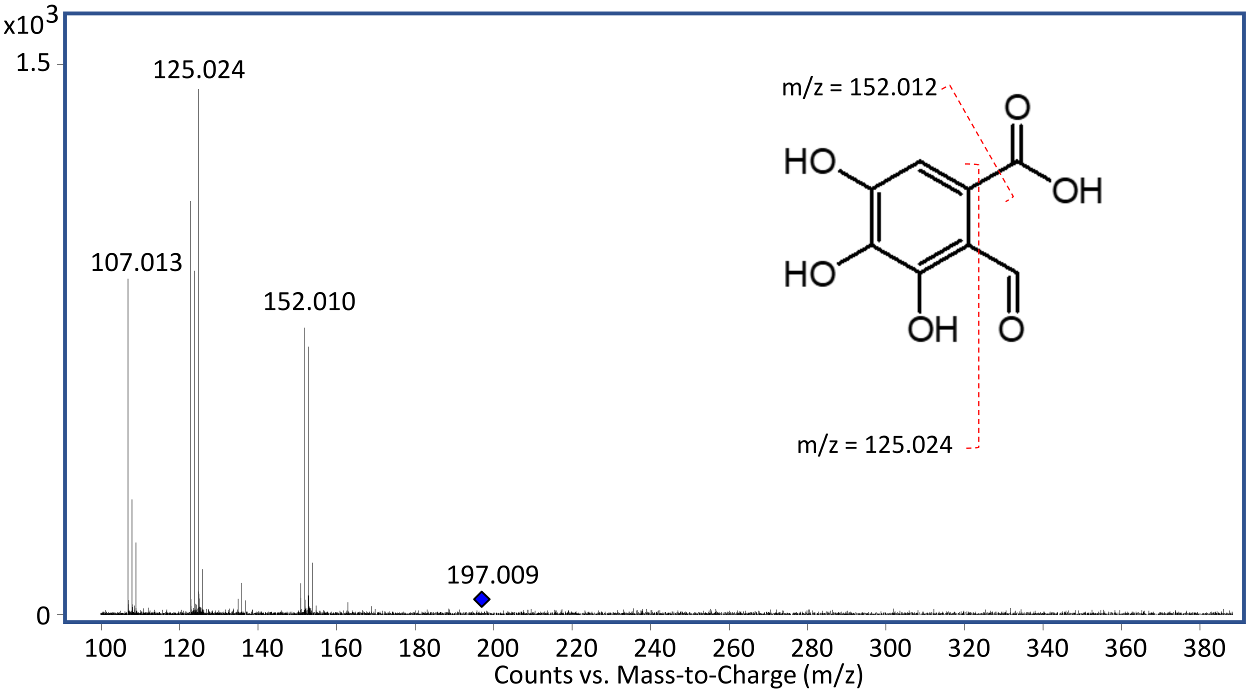

Figure S.5: Tandem mass spectrum of m_3 (t_R = 4.4 min, $C_8H_6O_6$) with hypothesised structure

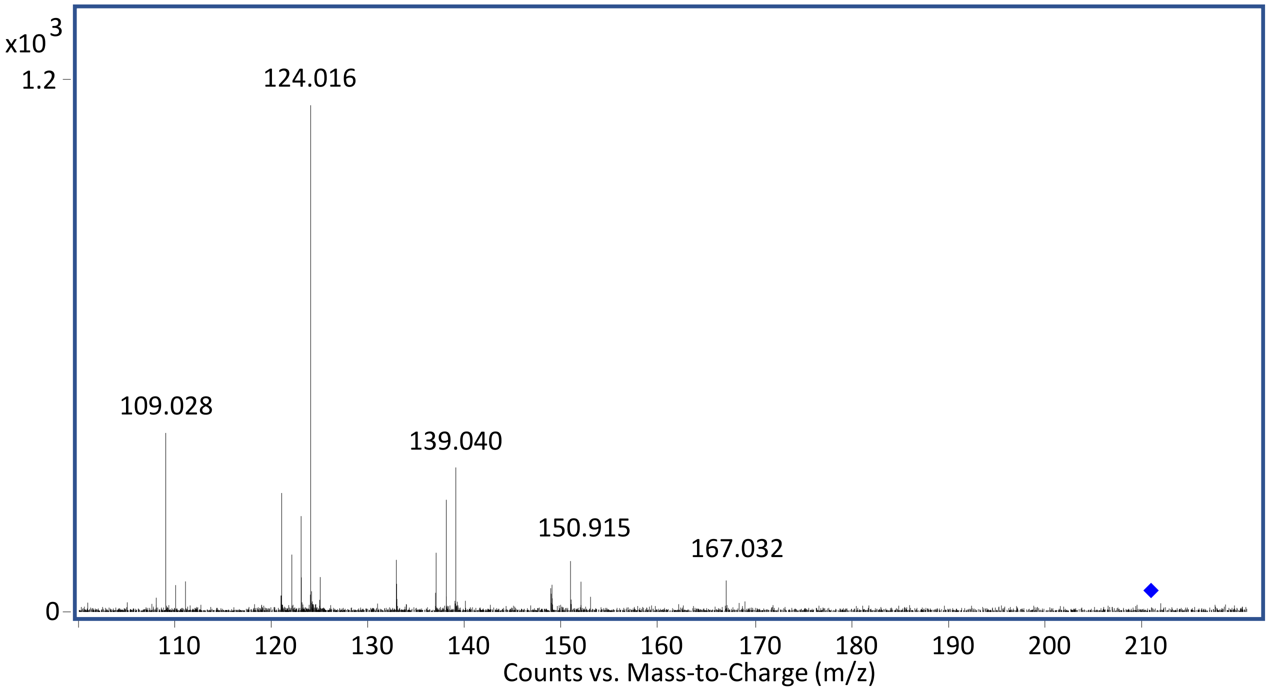

Figure S.6: Tandem mass spectrum of m_4 (t_R = 4.6 min, $C_9H_8O_6$)

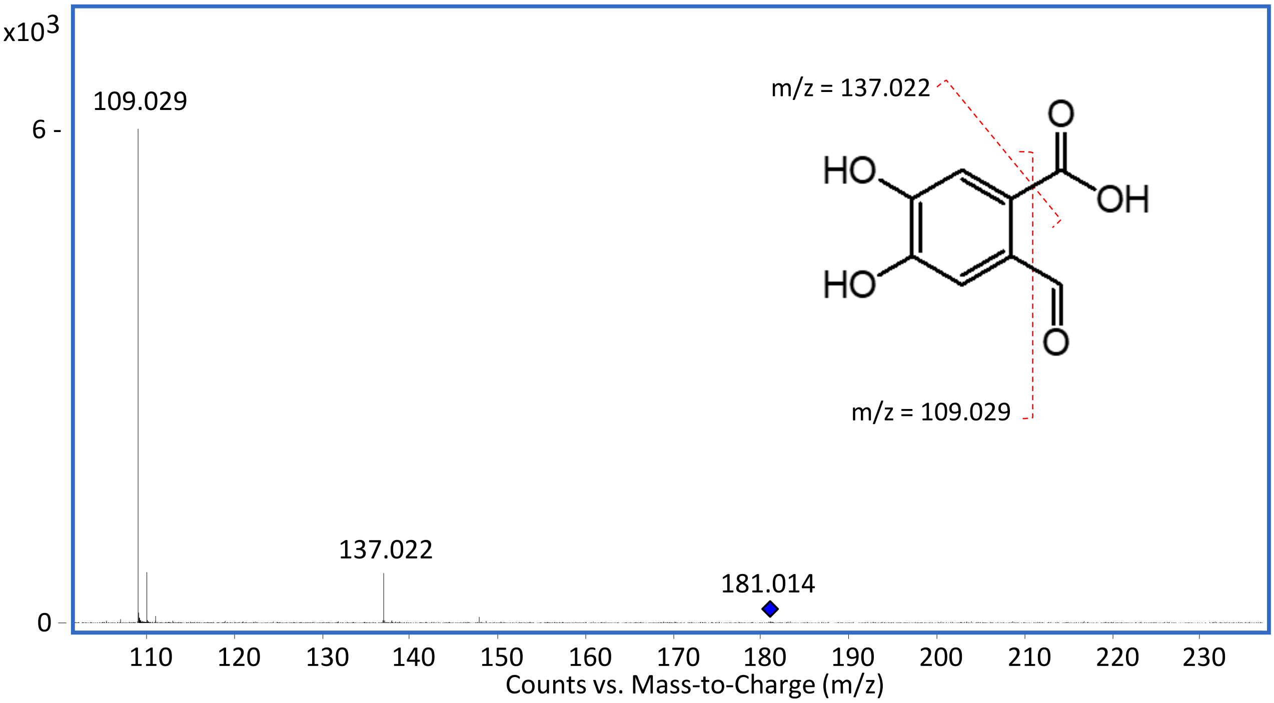

Figure S.7: Tandem mass spectrum of m_5 (t_R = 6.2 min, $C_8H_6O_5$) with hypothesised structure

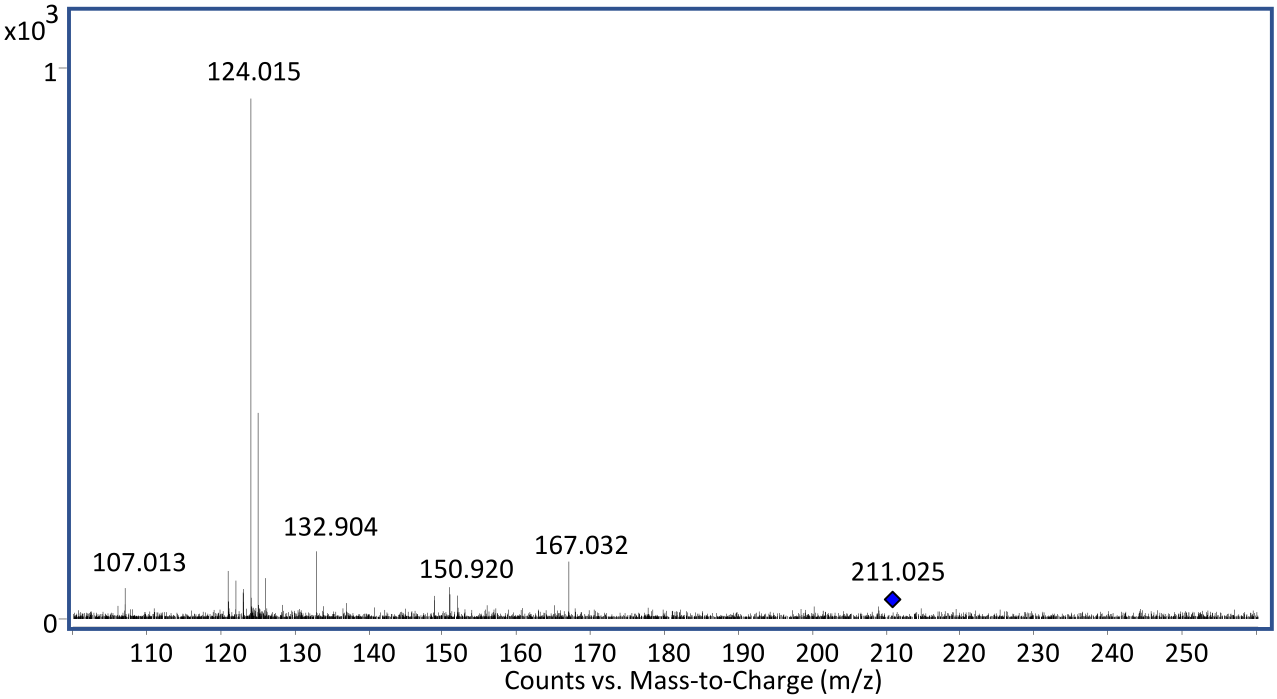

Figure S.8: Tandem mass spectrum of m_6 (t_R = 7.3 min, $C_9H_8O_6$)

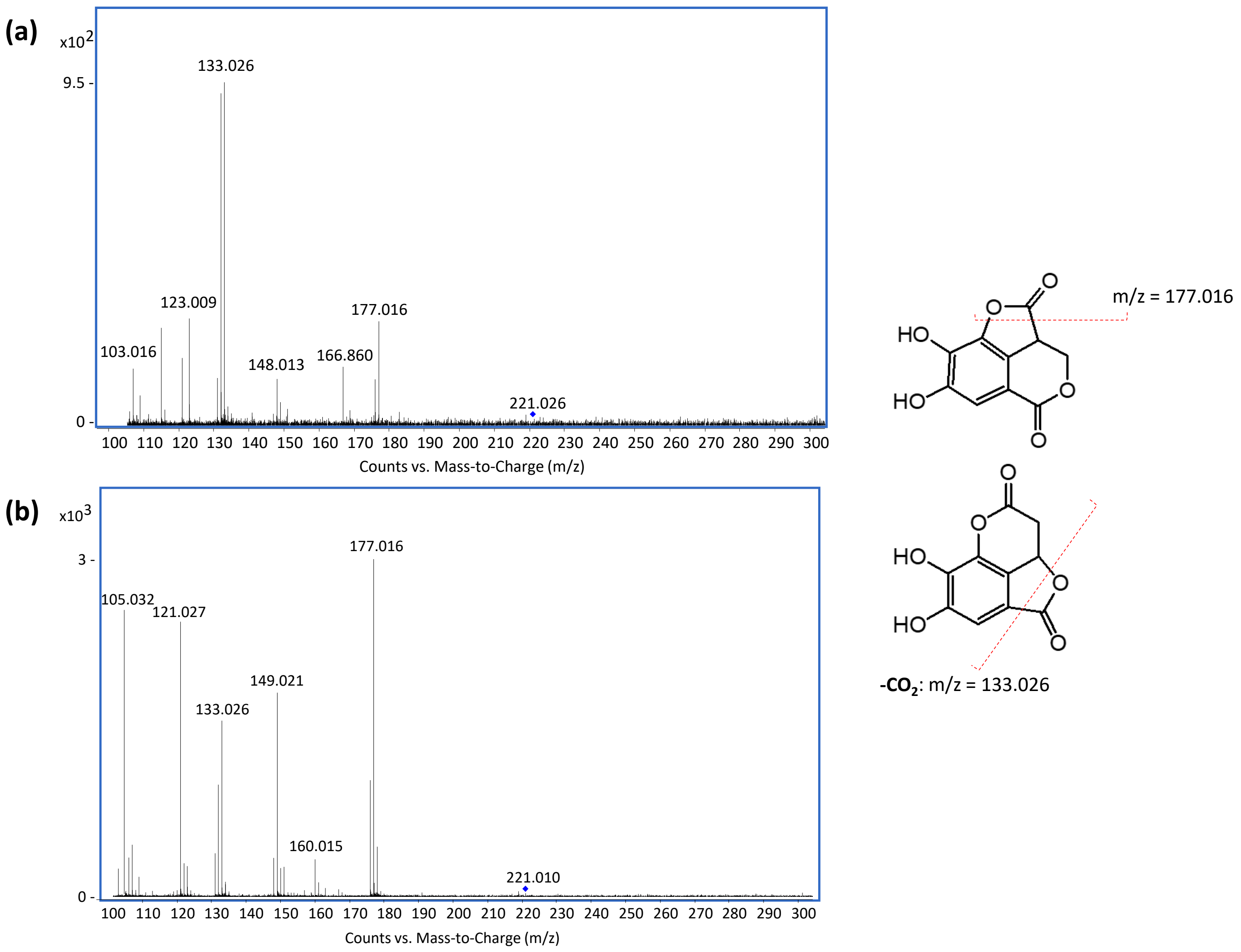

Figure S.9: Tandem mass spectrum of m_7 (spectrum a, t_R = 8.2 min, $C_9H_8O_5$) and m_8 (spectrum b, t_R = 8.9 min, $C_9H_8O_5$) with hypothesised structures

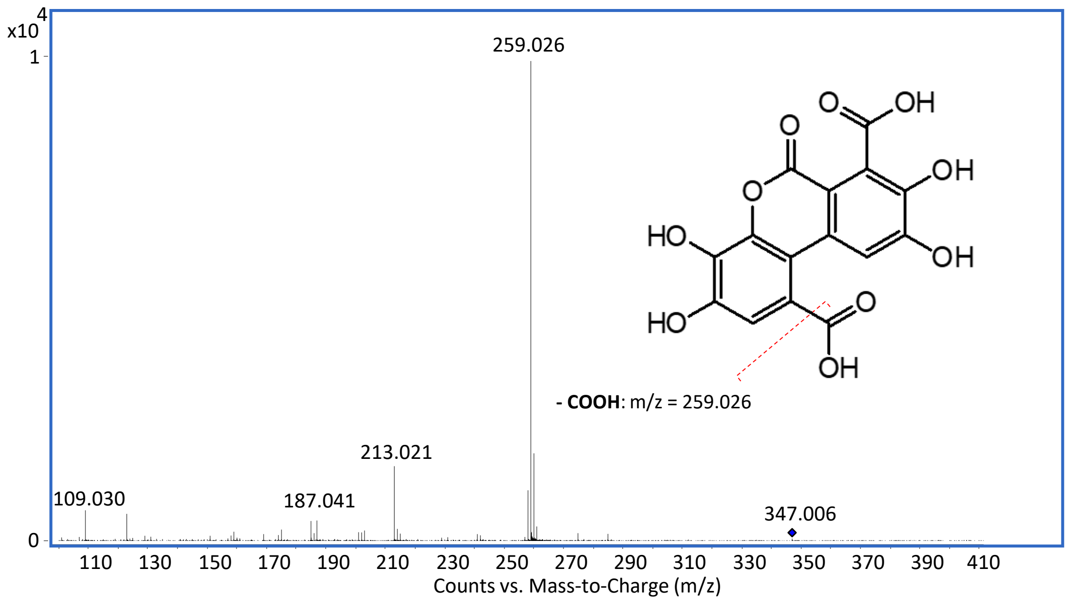

Figure S.10: Tandem mass spectra of m_9 (t_R = 9.5 min, $C_{15}H_8O_{10}$) with hypothesised structure

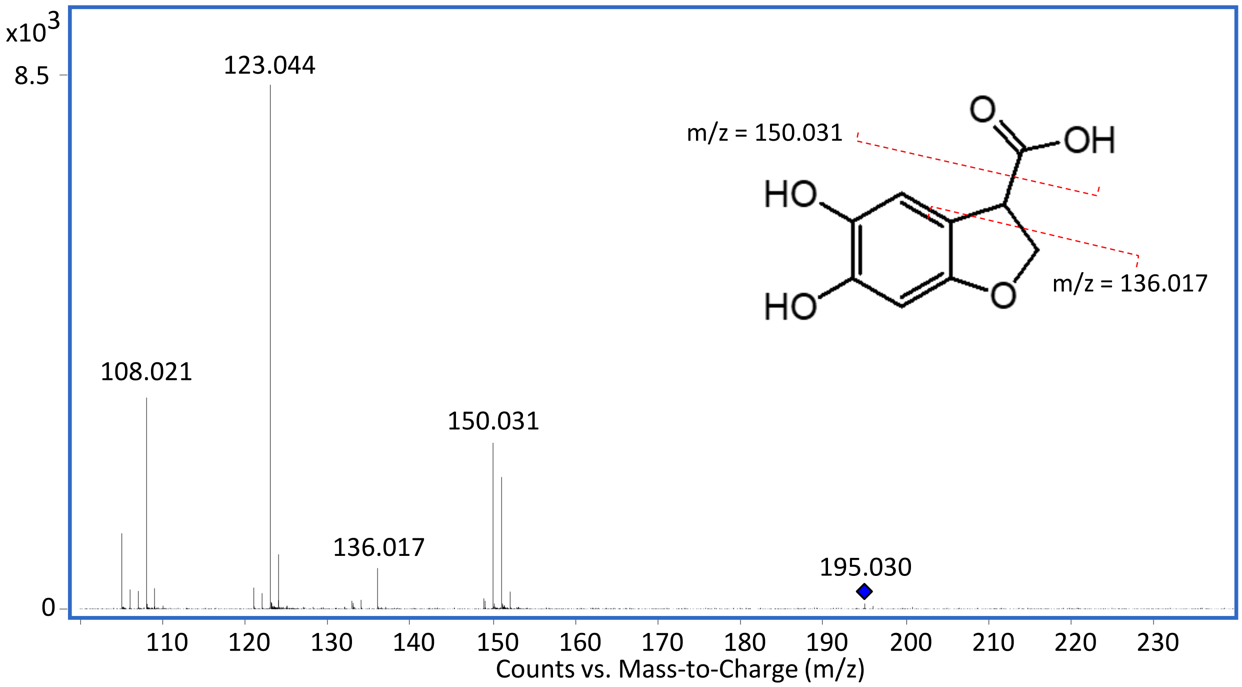

Figure S.11: Tandem mass spectrum of m_{10} (t_R = 9.7 min, $C_9H_8O_5$) with hypothesised structure

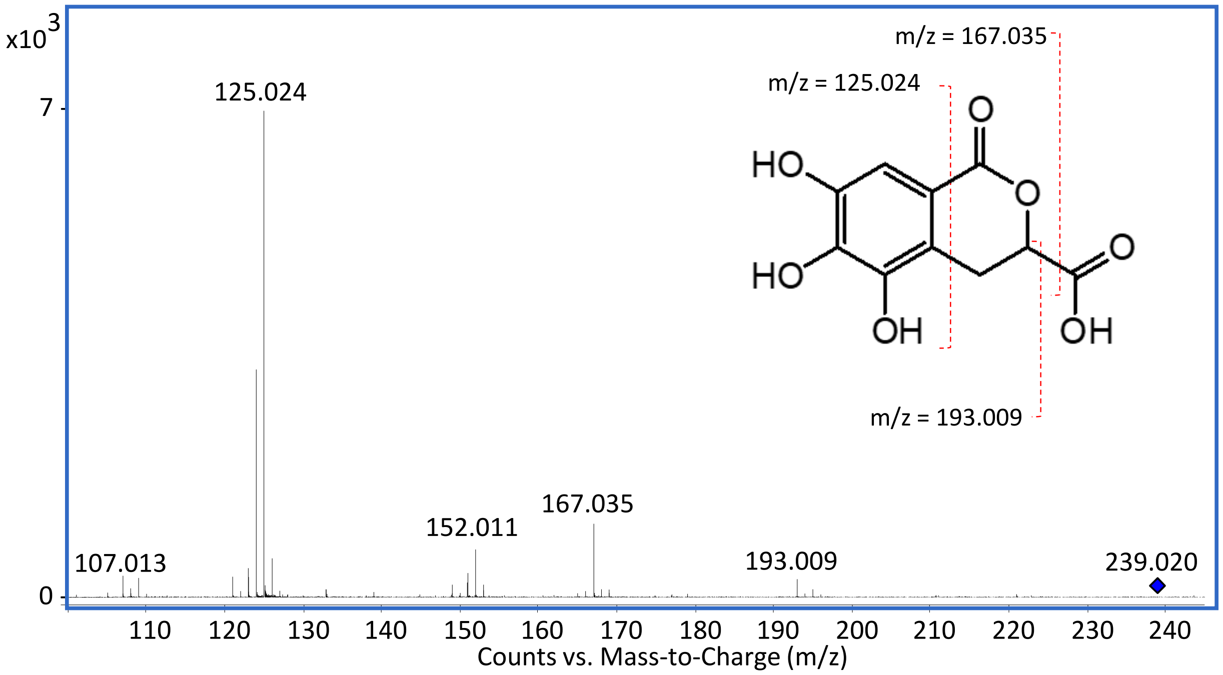

Figure S.12: Tandem mass spectrum of m_{11} (t_R = 9.9 min, $C_{10}H_8O_7$) with hypothesised structure

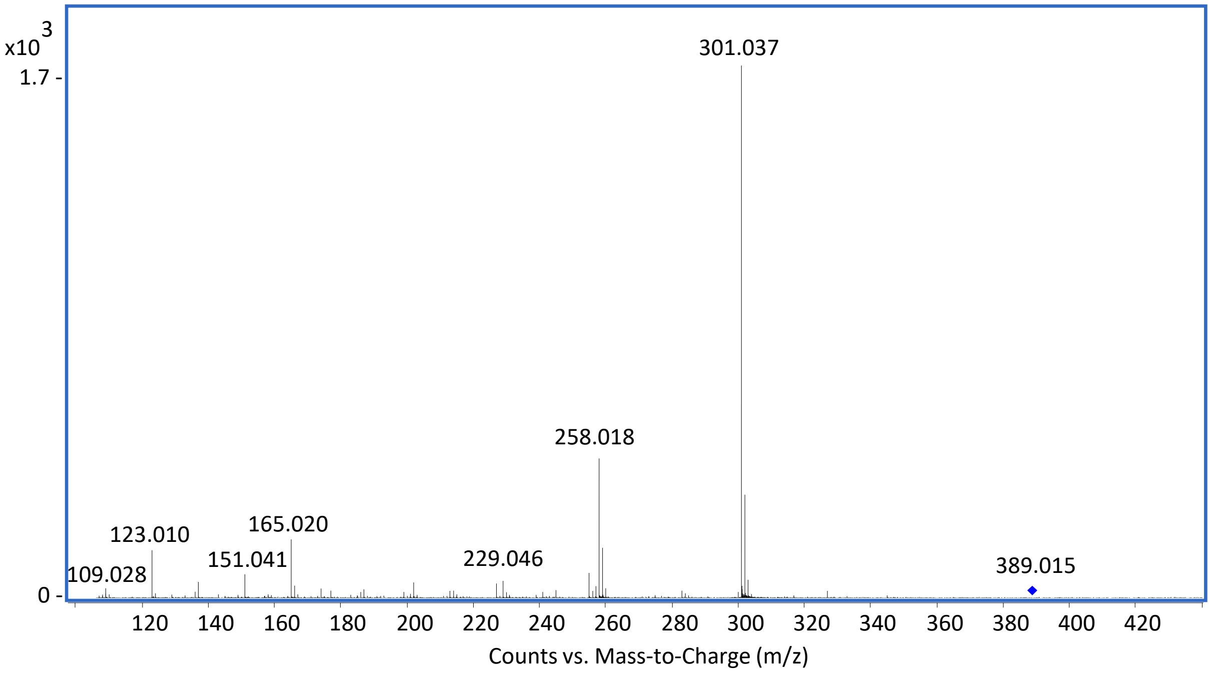

Figure S.13: Tandem mass spectrum of m_{12} (t_R = 10.5 min, $C_{17}H_{10}O_{11}$) with hypothesised structure

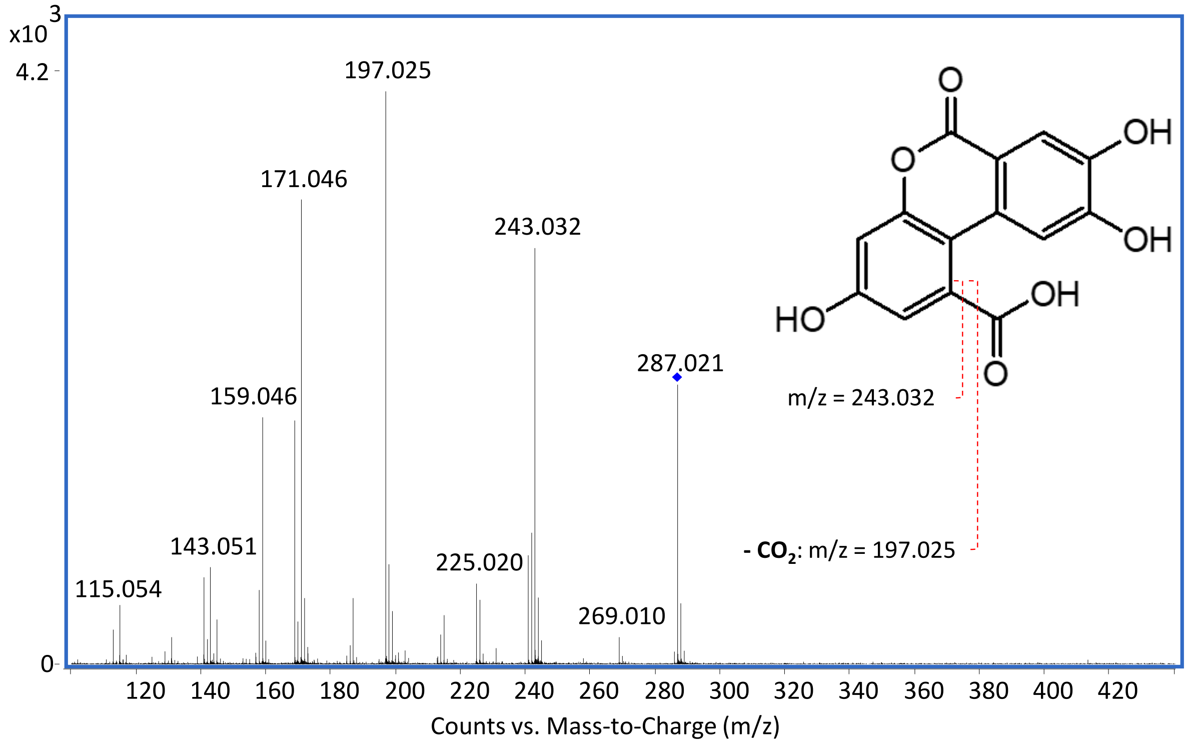

Figure S.14: Tandem mass spectrum of m_{13} (t_R = 12.3 min, $C_{14}H_8O_7$) with hypothesised structure

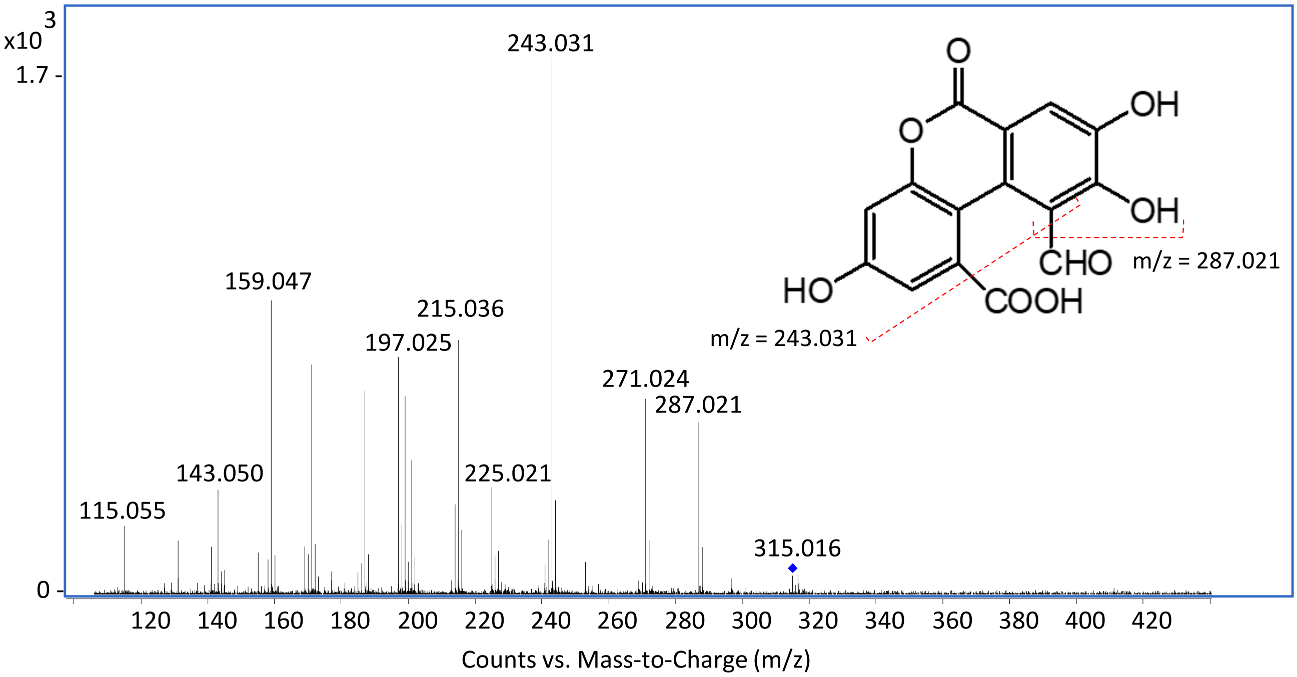

Figure S.15: Tandem mass spectrum of m_{14} (t_R = 13.2 min, $C_{15}H_8O_8$) with hypothesised structure
